# Association between the triglyceride to high-density lipoprotein cholesterol ratio and the risk of gestational diabetes mellitus: a second analysis based on data from a prospective cohort study

**DOI:** 10.3389/fendo.2023.1153072

**Published:** 2023-07-27

**Authors:** Yun You, Haofei Hu, Changchun Cao, Yong Han, Jie Tang, Weihua Zhao

**Affiliations:** ^1^ Department of Obstetrics, Shantou University Medical College, Shantou, Guangdong, China; ^2^ Department of Obstetrics, Shenzhen Second People’s Hospital, Shenzhen, Guangdong, China; ^3^ Department of Nephrology, Shenzhen Second People’s Hospital, Shenzhen, Guangdong, China; ^4^ Department of Rehabilitation, Shenzhen Dapeng New District Nan’ao People’s Hospital, Shenzhen, Guangdong, China; ^5^ Department of Emergency, Shenzhen Second People’s Hospital, Shenzhen, Guangdong, China

**Keywords:** sensitivity analysis, logistic models, ROC curve, triglyceride to high-density lipoprotein cholesterol, diabetes, gestational

## Abstract

**Background:**

Although there is strong evidence linking triglyceride to high-density lipoprotein cholesterol (TG/HDL-C) ratio to insulin resistance and diabetes mellitus, its clinical importance in pregnant women has not been well determined. This study sought to determine the connection between the TG/HDL-C ratio in the first trimester and the eventual onset of gestational diabetes mellitus (GDM).

**Methods:**

We performed a secondary analysis of open-access data from a prospective cohort study. This present study included 590 singleton pregnant women at 10-14 weeks who visited the outpatient clinics for prenatal checks and were recorded at Incheon Seoul Women’s Hospital and Seoul Metropolitan Government Seoul National University Boramae Medical Center in Korea. A binary logistic regression model, a series of sensitivity analyses, and subgroup analysis were used to examine the relationship between TG/HDL-C ratio and incident GDM. A receiver operating characteristic (ROC) analysis was also conducted to assess the ability of the TG/HDL-C ratio to predict GDM.

**Results:**

The mean age of the included individuals was 32.06 ± 3.80 years old. The mean TG/HDL-C ratio was 1.96 ± 1.09. The incidence rate of GDM was 6.27%. After adjustment for potentially confounding variables, TG/HDL-C ratio was positively associated with incident GDM (OR=1.77, 95%CI: 1.32-2.38, P=0.0001). Sensitivity analyses and subgroup analysis demonstrated the validity of the relationship between the TG/HDL-C ratio and GDM. The TG/HDL-C ratio was a good predictor of GDM, with an area under the ROC curve of 0.7863 (95% CI: 0.7090-0.8637). The optimal TG/HDL-C ratio cut-off value for detecting GDM was 2.2684, with a sensitivity of 72.97% and specificity of 75.05%.

**Conclusion:**

Our results demonstrate that the elevated TG/HDL-C ratio is related to incident GDM. The TG/HDL-C ratio at 10-14 weeks could help identify pregnant women at risk for GDM and may make it possible for them to receive early and effective treatment to improve their prognosis.

## Introduction

The most common metabolic disorder during pregnancy is gestational diabetes mellitus (GDM), which is defined as diabetes found in the second or third trimester that was previously unknown (1). Aggravating physiological alterations in glucose metabolism during pregnancy may contribute to GDM. 15% to 22% of pregnancies globally are afflicted by it, and its occurrence is rising (2). As one of the most prevalent pregnancy medical complications, GDM raises the risk of pregnancy complications and unfavorable perinatal outcomes, including pregnancy-induced hypertension, abortion, preeclampsia, premature delivery, premature rupture of membranes, large-for-gestational-age infants, and others. Additionally, it raises the mother’s chance of developing type 2 diabetes and affects the long-term metabolism of offspring (3, 4), posing a financial and public health burden.

It is a common phenomenon that maternal dyslipidemia during pregnancy is significantly higher than the physiological range (5). Hyperlipidemia is frequently found in the second half of pregnancy and is considered a biologically necessary mechanism to supply the fetus with fuel and nutrients (6). Early pregnancy causes a minor increase in lipid levels, whereas later pregnancy causes a considerable boost. Determining if a lipid rise is pathogenic or physiological might be challenging. The connection between lipid profiles and GDM is still up for debate. Although lipid levels during pregnancy have been extensively investigated, the findings are inconsistent (7). Some researchers confirm the significant increase in serum lipid profile, including concentrations of triglyceride to high-density lipoprotein cholesterol (TG/HDL-C) ratio and low-density lipoprotein cholesterol to high-density lipoprotein cholesterol (LDL-C/HDL-C) ratio in mothers with GDM compared to healthy pregnancies (8, 9). However, some studies have reported no significant differences in serum triglyceride (TG), total cholesterol (TC), low-density lipoprotein cholesterol (LDL-C), high-density lipoprotein cholesterol (HDL-C), and TG/HDL-C ratio between women with and without GDM (10, 11). Researchers have previously found a link between insulin resistance (IR), diabetes mellitus, and TG/HDL-C ratio (12, 13). However, few studies have also been done to determine whether TG/HDL-C ratio is linked to GDM and whether TG/HDL-C ratio in the first trimester can be used clinically to identify women at risk of GDM later. In the current study, we investigated whether early pregnancy TG/HDL-C ratio was associated with a later risk of developing gestational diabetes mellitus.

## Methods

### Data source

We downloaded the raw data freely from (https://journals.plos.org/plosone), provided by Lee SM et al. (14). From: Nonalcoholic fatty liver disease is a risk factor for large-for-gestational-age birthweight. The Creative Commons Attribution License, which allows unrestricted use, distribution, and reproduction in any format as long as the original author and source are credited, was used to publish this open-access research.

### Study population

The original study enrolled 663 singleton pregnant women presenting for prenatal care before 14 weeks of gestation at Incheon Seoul Women Hospital and Seoul Metropolitan Government Seoul National University Boramae Medical Center in Seoul, Korea from November 2014 to July 2016, from the ongoing ‘Fatty Liver in Pregnancy’ registry (ClinicalTrials.gov registration no. NCT02276144). Before enrollment, all participants provided written informed consent according to the original study (14). In a non-selective approach, the initial researchers gathered the subsequent cases. The initial researchers used untraceable codes to encrypt participant identity information to protect their privacy.

The Institutional Review Board of the Seoul Metropolitan Government Seoul National University Boramae Medical Center and the Public Institutional Review Board of the Ministry of Health and Welfare of Korea approved the research ethics (14). Therefore, there was no need for ethical approval of this secondary analysis. Also, the Declaration of Helsinki was followed in conducting the initial study.

**Figure 1:**
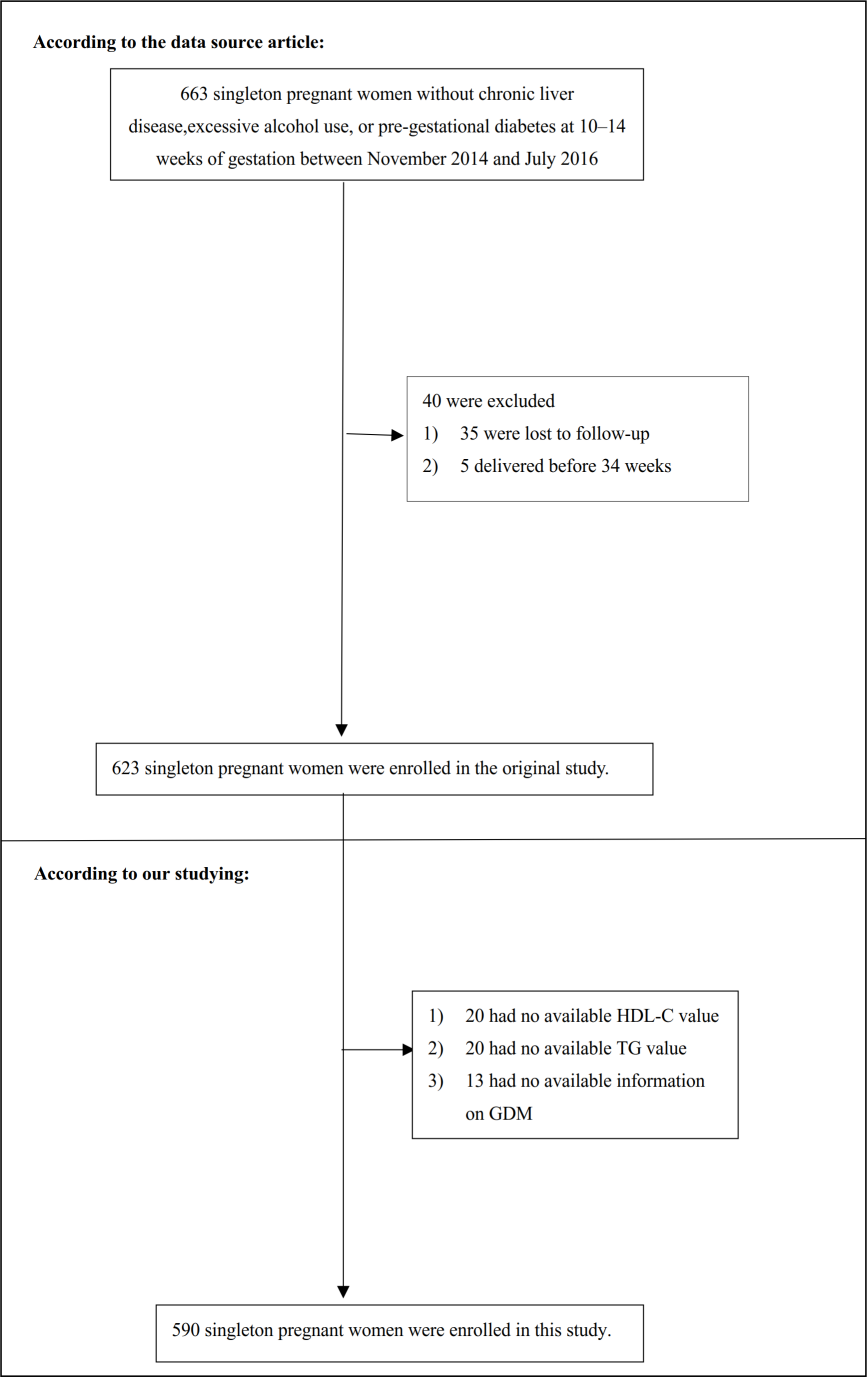
Flowchart of study participants. **Figure 1** showed the inclusion of participants. 623 participants were assessed for eligibility in the original study. We excluded patients with missing values of HDL-C (n=20), TG (n=20), and lack of information on GDM (n=13). The final analysis included 590 subjects in the present study.

Patients who (1): had underlying chronic liver disease, high alcohol consumption, or pre-gestational diabetes (2); were lost to follow-up; or (3) had a premature birth before 34 weeks were omitted from the final analysis. Consequently, the initial study contained 623 participants. In our present study, we further excluded missing values of HDL-C (n=20), TG (n=20), and lack of information on GDM (n=13). Finally, the present study included 590 eligible participants (**Figure 1**).

### Variables

#### TG/HDL-C ratio

At 10–14 weeks gestation, an automated analyzer was used to measure the levels of HDL-C and TG in venous blood after fasting for at least 8 hours. [serum TG (mmol/L)]/[serum HDL-C (mmol/L)] was the formula used to calculate the TG/HDL-C ratio in detail.

#### Diagnosis of incident GDM

In accordance with the advice of the American College of Obstetricians and Gynecologists, all subjects were evaluated for the existence of GDM using the two-step method at 24-28 weeks (15). Serum glucose levels were assessed for the 50 g oral glucose challenge screening test (GCT) 1 hour following a 50 g oral glucose load in a non-fasting state. 7.8 mmol/l of serum glucose was considered to be a positive GCT. Those with a positive screening GCT underwent a follow-up 100 g oral glucose tolerance test. Two or more increased glucose levels-5.3 mmol/L for fasting glucose, 10 mmol/L for one hour, 8.6 mmol/L for two hours, and 7.8 mmol/L for three hours-were necessary for the diagnosis of GDM (16).

#### Covariates

The original study, our clinical experience, and previous studies on risk factors for GDM were all taken into consideration when choosing the variables for this investigation. Accordingly, the following variables were utilized as covariates based on the aforementioned concepts: (1) categorical variables: parity, hepatic steatosis; (2) continuous variables: age, pre-pregnancy body mass index (BMI), fasting plasma glucose (FPG), insulin, homeostasis model assessment-insulin resistance (HOMA-IR), alanine aminotransferase (ALT), adiponectin, aspartate aminotransferase (AST), TC, gamma-glutamyl transferase (GGT), LDL-C.

General clinical and demographic information was collected, including maternal age, parity, pre-gestational diabetes, a prior history of GDM, pre-gestational weight, height, alcohol consumption during pregnancy using the validated cut-annoyed-guilty-eye questionnaire, and a history of chronic liver diseases such as hepatitis B or hepatitis C, primary biliary cholangitis, autoimmune hepatitis, hemochromatosis, primary sclerosing cholangitis and Wilson’s disease (14). At 10-14 weeks gestation, a venous blood sample was collected to measure hematological markers such as TC, TG, ALT, AST, GGT, FPG, insulin, and adiponectin after fasting for at least 8 hours. [FPG (mmol/L)×insulin (μIU/mL)/22.5] was the formula used to calculate HOMA-IR in detail (17). As in previous studies, a semiquantitative grading system (grades 0-3) was used to determine the severity of hepatic steatosis (18).

### Statistical analysis

We first observed the distribution of baseline data based on tertiles of the TG/HDL-C ratio. The mean and standard deviation (SD) or median and quartile ranges (25th-75th percentile) were displayed for continuous variables, while frequencies and percentages were used to represent categorical variables. To examine differences between TG/HDL-C ratio groups, the one-way ANOVA, Kruskal Wallis H test, and the chi-square test were used. Incidence rates were expressed in cumulative incidence (19).

We created three models using univariate and multivariate logistic regression, including a non-adjusted model (Crude model: no covariates were adjusted), a model with minimal adjustments (Model I: only sociodemographic variables, such as age, pre-pregnancy BMI, and parity were adjusted), and a model with complete adjustments (Model II: covariates presented in **Table 1** were adjusted, including age, pre-pregnancy BMI, parity, hepatic steatosis, AST, GGT, ALT, TC, LDL-C, HOMA-IR, and adiponectin). Adjusted odds ratios (OR) with a 95% confidence interval (CI) were estimated to evaluate the risk of GDM. The OR changed by at least 10% after the covariance was included in the model; hence, the covariance should be adjusted (20).

**Table 1:** The Baseline Characteristics of participants.

TG/HDL-C ratio	T1(≤1.41)	T2(1.41 to ≤2.11)	T3(>2.11)	P-value
**Participants**	197	196	197	
**Age(years)**	31.55 ± 3.56	32.46 ± 3.56	32.18 ± 4.19	0.051
**Pre-pregnancy BMI (kg/m^2^)**	21.24 ± 2.96	22.05 ± 3.56	22.82 ± 3.77	<0.001
**Parity**				0.050
**No**	117 (59.39%)	93 (47.45%)	100 (50.76%)	
**Yes**	80 (40.61%)	103 (52.55%)	97 (49.24%)	
**Hepatic steatosis**				<0.001
**Grade 0**	173 (87.82%)	167 (85.20%)	139 (70.56%)	
**Grade 1**	23 (11.68%)	23 (11.73%)	39 (19.80%)	
**Grade 2**	1 (0.51%)	4 (2.04%)	13 (6.60%)	
**Grade 3**	0 (0.00%)	2 (1.02%)	6 (3.05%)	
**HDL-C (mg/dL)**	74.09 ± 11.78	64.84 ± 10.90	55.82 ± 11.22	<0.001
**TG (mg/dL)**	81.88 ± 19.20	110.81 ± 21.68	164.24 ± 49.37	<0.001
**TC (mg/dL)**	171.14 ± 26.68	172.08 ± 26.35	175.36 ± 28.46	0.271
**LDL-C (mg/dL)**	80.68 ± 21.20	85.09 ± 20.06	86.31 ± 23.61	0.026
**ALT (IU/L)**	11 (8-14)	11 (8-15)	12 (8-18)	<0.001
**AST (IU/L)**	16 (14-18)	16 (14-19)	17 (14-21)	0.036
**GGT(IU/L)**	11 (9-14)	12 (10-15)	13 (10-17)	0.022
**FPG ((mg/dL)**	76.93 ± 10.25	76.88 ± 8.59	77.29 ± 10.29	0.903
**Insulin (μIU/mL)**	6.40 (4.27-9.53)	7.90 (5.50-10.90)	10.70 (7.50-15.30)	<0.001
**HOMA-IR**	1.59 ± 2.42	1.74 ± 1.13	2.34 ± 1.48	<0.001
**Adiponectin (ng/mL)**	7602.06 ± 4979.12	6234.51 ± 3705.93	4337.39 ± 3374.96	<0.001
**TG/HDL-C ratio**	1.11 ± 0.22	1.71 ± 0.20	3.05 ± 1.23	<0.001

Values were n(%) or mean ± SD or or median (quartile).

TG/HDL-C ratio, triglyceride to high-density lipoprotein cholesterol ratio; BMI, body mass index; ALT, alanine aminotransferase; AST, aspartate aminotransferase; GGT, gamma-glutamyl transferase; HDL-C, high-density lipoprotein cholesterol; TC, total cholesterol; TG, triglycerides; LDL-C, low-density lipid cholesterol; FPG, fasting plasma glucose; HOMA-IR, homeostasis model assessment-insulin resistance.

We conducted a number of sensitivity analyses to evaluate how reliable our findings were. To test the results of the TG/HDL-C ratio as a continuous variable and investigate the likelihood of non-linearity, we turned the TG/HDL-C ratio into a categorical variable based on the tertile and calculated the P for the trend. Obese and nonalcoholic fatty liver disease was linked to a higher incidence of GDM (19, 21). To investigate the link between the TG/HDL-C ratio and GDM risk, we thus excluded people with pre-pregnancy BMI≥24kg/m^2^ or nonalcoholic fatty liver disease (grade of hepatic steatosis>0) in other sensitivity analyses. Besides, to ensure the robustness of the findings, we additionally added the continuity covariate as a curve to the equation (Model III) using a generalized additive model (GAM) (22). Further, by computing E-values, we investigated the possibility of unmeasured confounding between TG/HDL-C and GDM risk (23).

A stratified logistic regression model was used for the subgroup analysis across multiple subgroups (age, pre-pregnancy BMI, parity, hepatic steatosis, HOMA-IR). Firstly, continuous variable age (<35, ≥35 years) (24), pre-pregnancy BMI (<24, ≥24 kg/m2) (25), HOMA-IR FPG(≤2, >2) (26) were converted to a categorical variable based on the clinical cut point. Secondly, in addition to the stratification factor itself, we adjusted each stratification for all factors (age, pre-pregnancy BMI, parity, hepatic steatosis, AST, GGT, ALT, TC, LDL-C, HOMA-IR, adiponectin). Lastly, the likelihood ratio test of models with and without interaction terms was used to test for interactions (27).

Furthermore, we conducted receiver operating characteristic (ROC) analysis to evaluate the predictive ability of the TG/HDL-C ratio to GDM. We then calculated the area under the curve (AUC) of the ROC and the best cut-off point.

We used PASS15.0 for the sample size calculation. The sample size is calculated with reference to the preliminary study and based on the parameters, including power, Alpha, incidence rate, and odds ratios (28). The final sample size was calculated to require at least 106 cases. And a total of 590 participants were included in this study, which could satisfy the sample size requirement.

All the analyses in our study were performed with the statistical software package R (http://www.R-project.org, The R Foundation) and Empower-Stats (http://www.empowerstats.com, X&Y Solutions, Inc., Boston, MA). All tests were two-sided, and P < 0.05 was considered statistically significant.

## Results

### Characteristics of participants

In this study, 590 women without pre-gestational diabetes were enrolled. The average age was 32.06 ± 3.80 years. 37 women (6.27%) developed GDM at 24-28 weeks of gestation.

**Table 1** presents the baseline characteristics of the population. The TG/HDL-C ratio was divided into three groups according to the tertiles (T1 ≤ 1.41; 1.41<T2 ≤2.11; T3>2.11). We found that in the T3 group, participants generally had higher levels of pre-pregnancy BMI, LDL-C, TG, ALT, GGT, AST, insulin, HOMA-IR, and higher rates of grade 3 hepatic steatosis. In contrast, participants in the T3 group had lower levels of HDL-C and Adiponectin.

### The incidence rate of GDM

**Table 2** displays the cumulative incidence rate of GDM. The cumulative incidence rate of GDM in the overall women and three TG/HDL-C ratio groups were specifically 6.27% (4.31%-8.23%), 1.52% (0.20%-3.25%), 3.57% (0.95%-6.19%), and 13.71% (8.86%-18.55%). Compared with the T1 group, participants in T3 had a higher incidence rate of GDM (P<0.001 for trend).

**Table 2:** Incidence rate of incident gestational diabetes mellitus.

TG/HDL-C ratio	Participants(n)	GDM events(n)	Cumulative incidence rate (95% CI) (%)
**Total**	590	37	6.27 (4.31-8.23)
**T1**	197	3	1.52 (0.20-3.25)
**T2**	196	7	3.57 (0.95-6.19)
**T3**	197	27	13.71 (8.86-18.55)
**P for trend**			<0.001

TG/HDL-C ratio, triglyceride to high-density lipoprotein cholesterol ratio; CI, confidence interval; GDM, gestational diabetes mellitus.

### The results of univariate analyses using a binary logistic regression model

The results of the univariate analysis were shown in **Table 3**. The univariate analysis showed that pre-pregnancy BMI, grade of liver steatosis, TG, ALT, GGT, FPG, insulin, HOMA-IR, and TG/HDL-C ratio were positively associated with incident GDM. We also found that HDL-C was inversely associated with incident GDM.

**Table 3:** The results of the univariate analysis.

	Statistics	OR (95% CI)	*P* value
Participants			
**Age (years)**	32.06 ± 3.80	1.04 (0.95, 1.13)	0.4304
**Pre-pregnancy BMI (kg/m^2^)**	22.03 ± 3.50	1.28 (1.18, 1.39)	<0.0001
Parity			
**No**	310 (52.54%)	ref	
**Yes**	280 (47.46%)	1.05 (0.54, 2.05)	0.8809
Hepatic steatosis			
**Grade 0**	479 (81.19%)	ref	
**Grade 1**	85 (14.41%)	3.43 (1.46, 8.03)	0.0046
**Grade 2**	18 (3.05%)	28.94 (10.13, 82.68)	<0.0001
**Grade 3**	8 (1.36%)	17.36 (3.81, 79.04)	0.0002
**HDL-C (mg/dL)**	64.91 ± 13.54	0.97 (0.94, 0.99)	0.0094
**TG (mg/dL)**	118.99 ± 47.51	1.02 (1.01, 1.03)	<0.0001
**TC (mg/dL)**	172.86 ± 27.19	1.01 (1.00, 1.02)	0.0645
**LDL-C (mg/dL)**	84.03 ± 21.77	1.00 (0.99, 1.02)	0.9387
**ALT (IU/L)**	13.42 ± 9.58	1.04 (1.01, 1.06)	0.0017
**AST (IU/L)**	17.82 ± 8.10	1.02 (0.99, 1.05)	0.1354
**GGT(IU/L)**	14.04 ± 8.64	1.04 (1.01, 1.07)	0.0020
**FPG ((mg/dL)**	77.03 ± 9.73	1.07 (1.04, 1.10)	<0.0001
**Insulin (μIU/mL)**	9.56 ± 6.67	1.12 (1.07, 1.17)	<0.0001
**HOMA-IR**	1.89 ± 1.79	1.48 (1.22, 1.78)	<0.0001
**Adiponectin (ng/mL)**	6057.69 ± 4287.78	1.00 (1.00, 1.00)	<0.0001
**TG/HDL-C ratio**	1.96 ± 1.09	2.24 (1.68, 2.98)	<0.0001

Values are n(%) or mean ± SD.

TG/HDL-C ratio, triglyceride to high-density lipoprotein cholesterol ratio; BMI, body mass index; ALT, alanine aminotransferase; AST, aspartate aminotransferase; GGT, gamma-glutamyl transferase; HDL-C, high-density lipoprotein cholesterol; TC, total cholesterol; TG, triglycerides; LDL-C, low-density lipid cholesterol; FPG, fasting plasma glucose; HOMA-IR, homeostasis model assessment-insulin resistance.

### The results of multivariate analyses using the binary logistic regression model

**Table 4** showed that the binary logistic regression model was used to evaluate the association between TG/HDL-C ratio and incident GDM. In the non-adjusted model (Crude model), TG/HDL-C ratio showed a positive association with incident GDM (OR: 2.24, 95%: 1.68-2.98, P <0.0001). When only demographic factors were taken into account in the minimally-adjusted model (Model I), the risk of GDM increased by 1.10 times for every additional unit of the TG/HDL-C ratio (OR= 2.10, 95%: 1.55-2.85, P <0.0001). In the fully-adjusted model (Model II), each additional unit of TG/HDL-C ratio was accompanied by a 77% increase in GDM risk (OR=1.77, 95%CI: 1.32-2.38, P=0.0001). The results were statistically significant.

**Table 4:** Relationship between TG/HDL-C ratio and the incident GDM in different models.

Variable	Crude model (OR.,95% CI, P)	Model I (OR,95% CI, P)	Model II (OR,95% CI, P)	Model III (OR,95% CI, P)
**TG/HDL-C ratio**	2.24 (1.68, 2.98) <0.0001	2.10 (1.55, 2.85) <0.0001	1.77 (1.32, 2.38) 0.0001	1.85 (1.35, 2.52) 0.0001
TG/HDL-C ratio (tertile)				
**Q1**	Ref.	Ref.	Ref.	Ref.
**Q2**	2.40 (0.61, 9.40) 0.2104	1.86 (0.45, 7.64) 0.3922	2.00 (0.41, 9.71) 0.3902	1.41 (0.27, 7.35) 0.6849
**Q3**	10.27 (3.06, 34.44) 0.0002	7.54 (2.17, 26.23) 0.0015	4.38 (1.05, 18.29) 0.0429	4.75 (1.12, 20.07) 0.0341
**P for trend**	<0.0001	0.0001	0.0202	0.0108

Crude model: we did not adjust other covariants.

Model I: we adjusted age, pre-pregnancy BMI, parity.

Model II: we adjusted age, pre-pregnancy BMI, parity, hepatic steatosis, AST, GGT, ALT, TC, LDL-C, HOMA-IR, adiponectin.

Model III: we adjusted age(smooth), pre-pregnancy BMI(smooth), parity, hepatic steatosis, AST(smooth), GGT(smooth), ALT(smooth), TC(smooth), LDL-C(smooth), HOMA-IR(smooth), adiponectin(smooth).

HR, Hazard ratios; CI: confidence, Ref: reference; eGFR, evaluated glomerular filtration rate(mL/min·1.73 m2); NAFLD, non-alcoholic fatty liver disease.

OR, odds ratios; CI, confidence interval; Ref, Reference; TG/HDL-C ratio, triglyceride to high-density lipoprotein cholesterol ratio.

### Sensitive analysis

We used a number of sensitivity analyses to evaluate the robustness of our findings. We treated the TG/HDL-C ratio as a categorical variable and then reintroduced the categorical-transformed TG/HDL-C ratio into the model. After transforming the TG/HDL-C ratio into a categorical variable, the results showed that the trends in effect sizes (OR) between groups were equidistant. P for the trend matched the findings when TG/HDL-C ratio was continuous. Moreover, a GAM added the continuity covariate to the equation. We discovered that the GAM model’s results aligned with the fully adjusted model (OR=1.85, 95%CI: 1.35-2.52, P=0.0001) (**Table 4**). Besides, this study also produced E-values to assess the influence of possible unmeasured confounding between the TG/HDL-C ratio and GDM risk. The E value for this study was 2.94. The E-value was higher than the relative risk of TG/HDL-C ratio and unmeasured confounders, indicating that the association between TG/HDL-C ratio and incident GDM was not significantly affected by unmeasured or unknown confounders.

In addition, we performed other sensitivity analyses on individuals with BMI<24kg/m^2^. There was also a positive relationship between the TG/HDL-C ratio and GDM risk after adjusting for confounding covariates (OR=1.88, 95%CI: 1.26-2.81) (**Table 5**). Moreover, we included individuals with grade 0 hepatic steatosis for other sensitivity analyses. The findings revealed that the TG/HDL-C ratio remained positively linked with the risk of GDM after adjusting for age, pre-pregnancy BMI, parity, AST, GGT, ALT, TC, LDL-C, HOMA-IR, adiponectin (OR= 2.06, 95%CI: 1.34-3.16) (**Table 5**). The sensitivity analysis suggested that our results were well-robust.

**Table 5:** Relationship between the TG/HDL-C ratio and incident GDM in different sensitivity analyses.

Exposure	Model I (OR,95%CI, P)	Model II (OR,95%CI, P)
**TG/HDL-C ratio**	1.88 (1.26, 2.81) 0.0018	2.06 (1.34, 3.16) 0.0010
TG/HDL-C ratio (tertile)		
**T1**	Ref.	Ref.
**T2**	0.35 (0.03, 4.18) 0.4101	0.80 (0.11, 6.06) 0.8312
**T3**	2.32 (0.43, 12.45) 0.3265	4.92 (1.00, 24.23) 0.0504
**P for trend**	0.1785	0.0150

Model I was sensitivity analysis after excluding those with pre-pregnancy BMI≥24kg/m^2^. We adjusted age, parity, hepatic steatosis, AST, GGT, ALT, TC, LDL-C, HOMA-IR, and adiponectin.

Model II was sensitivity analysis after including those with grade 0 hepatic steatosis. We adjusted age, pre-pregnancy BMI, parity, AST, GGT, ALT, TC, LDL-C, HOMA-IR, and adiponectin.

OR, odds ratios; CI, confidence; Ref, reference; TG/HDL-C ratio, triglyceride to high-density lipoprotein cholesterol ratio.

### The results of the subgroup analysis

Subgroup analysis was used to identify potential confounding factors that could have impacted the relationship between TG/HDL-C and the incident GDM (**Table 6**). Age, pre-pregnancy BMI, parity, hepatic steatosis, and HOMA-IR were chosen as stratification variables. The potential confounding variables mentioned above did not affect the relationship between TG/HDL-C ratio and GDM risk. The subgroup analysis showed that our results were well-robust.

**Table 6:** Effect size of TG/HDL-C ratio on GDM in prespecified and exploratory subgroups.

Characteristic	No of patients	Effect size(95%CI)	P value	P for interaction
**Age (years)**				0.7797
**<35**	453	1.96 (1.39, 2.75)	0.0001	
**≥35**	137	1.71 (0.70, 4.15)	0.2392	
**Pre-pregnancy BMI (kg/m^2^)**				0.9792
**<24**	457	1.78 (1.20, 2.66)	0.0046	
**≥24**	132	1.80 (1.07, 3.02)	0.0266	
**Parity**				0.3199
**No**	310	1.99 (1.32, 3.02)	0.0011	
**Yes**	280	1.46 (0.93, 2.31)	0.1010	
**Hepatic steatosis**				0.3922
**Grade 0**	479	2.03 (1.32, 3.12)	0.0012	
**Grade 1-3**	111	1.54 (0.98, 2.42)	0.0599	
**HOMA-IR**				0.2712
**≤2**	388	1.24 (0.59, 2.59)	0.5647	
**>2**	201	1.94 (1.32, 2.86)	0.0007	

Note 1: Above model adjusted for we adjusted for age, pre-pregnancy BMI, parity, hepatic steatosis, AST, GGT, ALT, TC, LDL-C, HOMA-IR, and adiponectin.

Note 2: The model is not adjusted for the stratification variable in each case.

### ROC analysis

**Figure 2:**
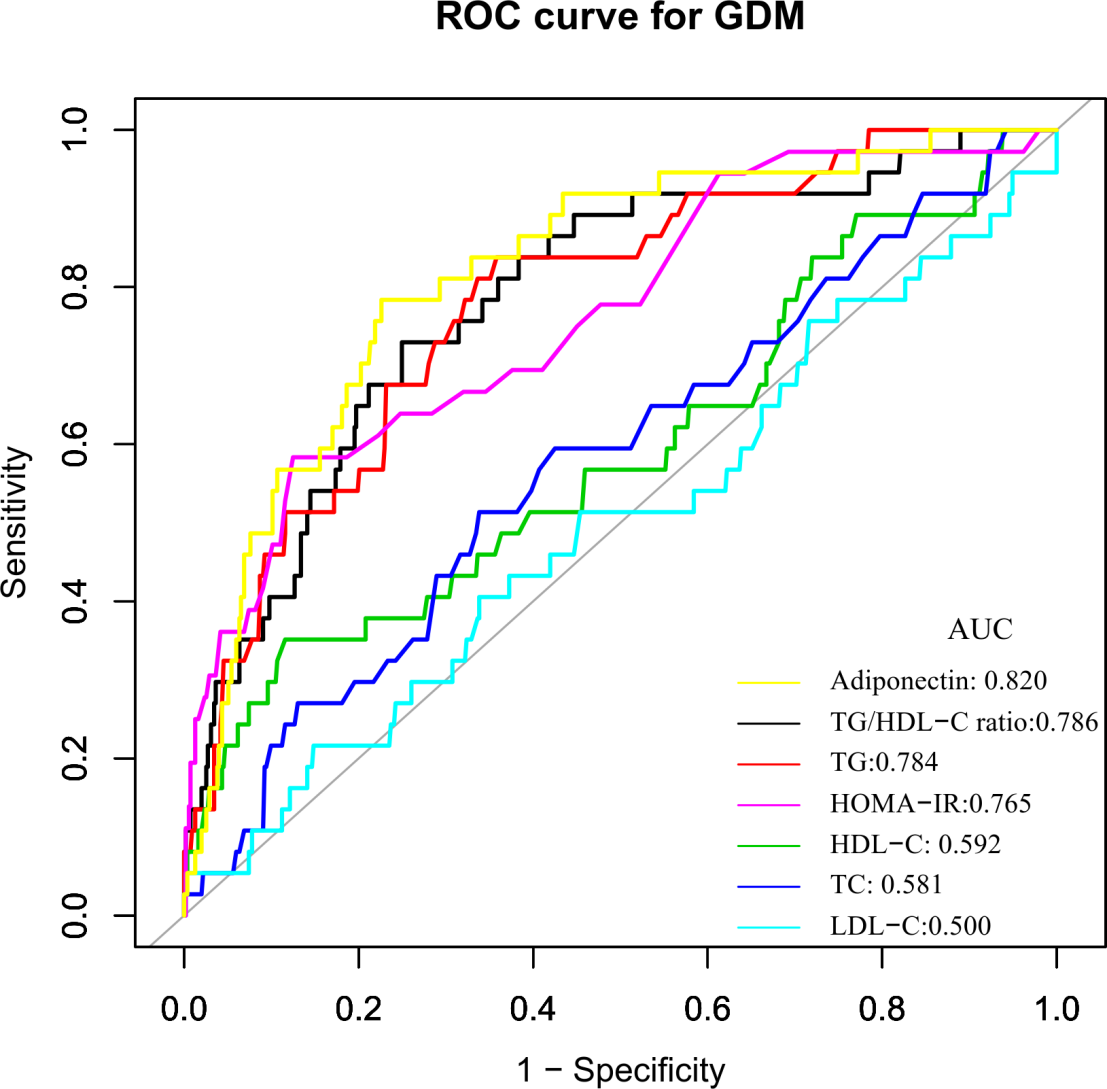
The TG/HDL-C ratio for predicting GDM in all participants by ROC analyses. ROC analysis was further conducted to explore the ability of the TG/HDL-C ratio to predict GDM. The results showed that the AUC of the TG/HDL-C ratio was 0.7863. Compared to TG, HDL-C, TC, LDL-C, FPG, insulin, and HOMA-IR, the AUC of the TG/HDL-C ratio for predicting DM was the highest.

ROC analysis was further conducted to explore the ability of the TG/HDL-C ratio to predict GDM. The results showed that the AUC of the TG/HDL-C ratio was 0.7863 (95%CI: 0.7090-0.8637) (**Table 7** and **Figure 2**). Compared to TG, HDL-C, TC, LDL-C, FPG, adiponectin, and HOMA-IR, the AUC of the TG/HDL-C ratio was predicted to be higher for DM. Youden’s index determined that 2.2684 was the optimal cut-off point for using the TG/HDL-C ratio to predict GDM, with matching specificity and sensitivity values of 75.05 and 72.97%.

**Table 7:** Areas under the Receiver operating characteristic curves for each evaluated parameters in identifying GDM.

Test	AUROC	95%CI	Best threshold	Specificity	Sensitivity	Youden Index
**TG/HDL-C ratio**	0.7863	0.7090-0.8637	2.2684	0.7505	0.7297	0.4802
**TG**	0.7837	0.7092-0.8582	121.5000	0.6420	0.8378	0.4798
**HDL-C**	0.5923	0.4869-0.6977	49.2000	0.8843	0.3514	0.2357
**TC**	0.5810	0.4826-0.6794	181.5000	0.6618	0.5135	0.1753
**LDL-C**	0.5000	0.3968-0.6032	105.5500	0.8517	0.2162	0.0679
**FPG**	0.6584	0.5545-0.7623	90.5000	0.9566	0.3056	0.2622
**Insulin**	0.7702	0.6826-0.8578	13.9000	0.8659	0.6216	0.4875
**Adiponectin**	0.8202	0.7516-0.8887	2973.8000	0.7740	0.7838	0.5578
**HOMA-IR**	0.7649	0.6788-0.8510	2.7500	0.8752	0.5833	0.4585

TG/HDL-C ratio, triglyceride to high-density lipoprotein cholesterol ratio; HDL-C, high-density lipoprotein cholesterol; TC, total cholesterol; TG, triglycerides; LDL-C, low-density lipid cholesterol; FPG, fasting plasma glucose; HOMA-IR, homeostasis model assessment-insulin resistance; AUROC, Areas under the Receiver operating characteristic curves.

## Discussion

In the present study, we investigated the association between the TG/HDL-C ratio and the risk of GDM in the Korean population. Our findings showed that TG/HDL-C ratio was positively correlated with the incident GDM. We also demonstrated that TG/HDL-C ratio could predict GDM accurately with an AUC of 0.7863 (0.7090-0.8637), and the optimal cut-off point of TG/HDL-C ratio for predicting GDM was 2.2684, with a sensitivity of 75.05% and specificity of 72.97%. The TG/HDL-C ratio was superior to TG, HDL-C, TC, LDL-C, and HOMA-IR for predicting GDM in the population. Thus, TG/HDL-C ratio could be an effective noninvasive method for predicting DM.

The incidence of GDM increased to 12.70% in the general Korean population in recent years (29). The incidence of GDM in the present study was 6.27%, lower than the reported level. Since this study excluded women with chronic liver disease, excessive alcohol consumption, or pre-gestational diabetes, which are risk factors for GDM (21). Therefore, the fact that research participants had a lower incidence of GDM than the general population was acceptable. It’s important to note that the incidence of GDM was still 6.27% in this population. It is still essential to aggressively search for additional potential risk factors for GDM.

In pregnant women, particularly in GDM pregnancies, a higher blood level of TG is typical. This may be related to oxidative stress, insulin resistance, and a relative lack of insulin secretion (30). According to some studies, hypertriglyceridemia, especially in the early stages of pregnancy, is linked to GDM and insulin resistance (31, 32). According to Enquobahrie et al. (33), the chance of developing GDM increases by 10% for every 20 mg/dl increase in TG concentration. Additionally, they showed that pregnant women with TG levels higher than 137 mg/dl had a 3.5-fold increased risk of having GDM (33). Furthermore, whether or not women have GDM, it has been demonstrated that the level of maternal TG has a solid and independent relationship with birth weight (34). The increased risk of macrosomia in pregnant women with hypertriglyceridemia has some pathophysiological causes. In the third trimester of pregnancy, there might be considerable variations in TG serum levels. Insulin sensitivity and lipoprotein lipase activity rise during the first trimester of pregnancy. In contrast, the third trimester of pregnancy sees a decrease in lipoprotein lipase activity due to an increase in insulin resistance. This condition is more common in GDM (35). Additionally, it has been found that a moderate increase in HDL-C concentration is a protective factor for GDM and that HDL-C levels in the blood are negatively correlated with GDM risk (36). Since TG/HDL-C ratio is an index that combines TG and HDL-C, it is related to GDM (37). In a prospective study involving 954 healthy pregnant women, after adjusting for age, history of diabetes in the first-degree family, and first trimester-body mass index, the relative risk of GDM in the top tertile of TG/HDL-C ratio was 3.87-folds of its risk in women in the bottom tertile (28). Another prospective study involving 202 healthy pregnant women found that the TG/HDL-C ratio was a risk factor for GDM when pregnant women were obese. When pregnant women are not obese, the TG/HDL-C ratio is not associated with GDM (38). Our study showed a positive association between TG/HDL-C ratio and GDM risk, which is consistent with previous studies. In addition, our research shows that compared with TG, HDL-C, TC, LDL-C, and HOMA-IR, TG/HDL-C ratio is the best predictor of GDM risk. At the same time, in the sensitivity analysis, we found that the relationship between TG/HDL-C ratio and GDM risk can still be detected in Korean women with BMI<24kg/m^2^ or grade 0 hereditary steatosis. Compared with previous studies, our study included a different study population. In addition, we adjusted more covariates, such as hepatic steatosis, AST, GGT, ALT, TC, LDL-C, HOMA-IR, and adiponectin, which are all risk factors for GDM. More importantly, we used sensitivity and subgroup analysis methods to verify further the correlation between TG/HDL-C ratio and GDM. In short, our results further confirm the positive correlation between TG/HDL-C ratio and GDM risk in participants with different BMI, age, and HOMA-IR levels. These efforts demonstrate the relationship’s stability between the TG/HDL-C ratio and GDM risk. Therefore, this study further extends the application of the relationship between the TG/HDL-C ratio and GDM to the population. The results provide a reference for the clinical intervention of the TG/HDL-C ratio to reduce the risk of GDM. Therefore, this assay has excellent clinical value. The findings of this research should be conducive to future studies on establishing a predictive model of GDM risk.

According to Wang et al. (30), the area under the ROC curve for TG/HDL-C to detect GDM was 0.617 (95%CI: 0.548-0.686). With an AUC of 0.664 (0.595–0.733), TG/HDL-C was also found to potentially identify GDM risk in 352 Chinese women in single-center research (37). The logarithm of the TG/HDL-C ratio in early pregnancy has been proposed by Santos-Weis et al. as a valuable index to identify pregnant women with minimal risk of GDM before 24 weeks of gestation. In addition, our research shows that compared with TG, HDL-C, TC, LDL-C, FPG, and HOMA-IR, TG/HDL-C ratio is a better predictor of GDM risk. Clinical studies have revealed that hypoadiponectinemia is a risk factor of GDM (39, 40). Although the AUC was slightly larger for adiponectin than for TG/HDL-c in predicting GDM, the difference was not statistically significant (P=0.4931). Besides, after adjusting the HOMA-IR and adiponectin, we found that the TG/HDL-C ratio is still related to gestational diabetes. In addition, adiponectin is not routinely used in clinical practice to screen for GDM compared to lipids. Therefore, the use of TG/HDL-C for predicting the risk of GDM remains of general clinical value. Abnormal TG/HDL-C ratio can be a timely warning of GBM risk in clinical settings. Since 2.2684 is the best cut-off point for predicting GDM using the TG/HDL-C ratio, its corresponding specificity and sensitivity values were 75.05% and 72.97%, respectively. From a therapeutic perspective, it makes sense to maintain the TG/HDL-C ratio below the cut-off point.

The mechanism behind the association between the TG/HDL-C ratio and GDM is unknown, but IR may be involved. In pregnant women, elevated estrogen levels and insulin resistance can boost the liver’s lipid synthesis (7). These modifications in fat metabolism point to a physiological change in pregnant women’s bodies that prioritizes lipid metabolism over glucose metabolism. Pregnant women employ lipids as a source of energy to preserve glucose for the growth and development of the fetus. Bile acids, steroid hormones, and embryonic cell membranes can all be produced thanks to lipids (41). Early in pregnancy, there is an increase in the production of blood lipids and lipids, mainly triglycerides, which raises the blood levels of free fatty acids. High free fatty acids may impair insulin sensitivity (42), creating a vicious cycle between high TG levels and IR, which may lead to impaired glucose tolerance and the development of diabetes (43). Reduced insulin secretion, decreased insulin sensitivity, and reduced AMP-activated protein kinase activity are all possible effects of low HDL-C levels on glucose homeostasis (44–47). In addition, studies have shown that β-arrestin may be associated with metabolic disorders and may play a key role in the development of GDM (48). In addition, after adjusting the HOMA-IR, we found that the TG/HDL-C ratio is still related to gestational diabetes, indicating that the TG/HDL-C ratio may have other possible mechanisms to cause diabetes in addition to causing insulin resistance.

Our study has several following advantages. First, residual confounding factors were minimized by using strict statistical adjustments. Second, sensitivity analyses were conducted to ensure the robustness of the results. It included transforming the TG/HDL-C ratio into a categorical variable, using a GAM to insert the continuity covariate into the equation as a curve, and reanalyzing the association between the TG/HDL-C ratio and GDM after including participants with BMI<24kg/m^2^ or grade 0 hereditary steatosis. Third, the present study conducted a subgroup analysis to assess other risk factors that might influence the connection between the TG/HDL-C ratio and GDM.

The present study does have certain restrictions. First, because the link between TG/HDL-C ratio and GDM may differ depending on ethnicity, the findings of our investigation should be verified in different ethnic groups. Second, because the present study is a secondary analysis, it is impossible to make adjustments for factors like uric acid, family history of diabetes, hypertension, and renal function that were not present in the initial dataset. The authors, however, determined that unmeasured confounders were unlikely to explain the data after calculating the E-value to assess the possible influence of unaccounted-for confounders. Third, the original study did not address preterm infants before 34 weeks and how TG and HDL-C fluctuate over time. Future designs of our investigation may include preterm infants before 34 weeks, capturing additional confounding variables and variations in TG and HDL-C during follow-up. We will also explore the external validity of our results in other populations.

## Conclusion

In summary, the current study suggests that an elevated TG/HDL-C ratio has an independent and positive relationship with the risk of incident GDM and could be used as a predictor for GDM in the Korean population. Thus, the aberrant TG/HDL-C ratio facilitates the identification of Korean people at high risk of developing GDM. This would assist physicians in the early planning and implementation of care methods. The TG/HDL-C ratio may be an important routine screening test for gestational diabetes in pregnant women.

## Data availability statement

The datasets presented in this study can be found in online repositories. The names of the repository/repositories and accession number(s) can be found in the article/**Supplementary Material**.

## Ethics statement

The studies involving human participants were reviewed and approved by the Institutional Review Board of the Seoul Metropolitan Government Seoul National University Boramae Medical Center and the Public Institutional Review Board of the Ministry of Health and Welfare of Korea. The patients/participants provided their written informed consent to participate in this study.

## Author contributions

YY and HH contributed to the study concept and design, researched and interpreted the data, and drafted the manuscript. CC and YH analyzed the data and reviewed the manuscript. JT and HH oversaw the project’s progress, contributed to the discussion and reviewed the manuscript. As guarantors, WZ, JT, and HH had full access to all study data and were responsible for its integrity and accuracy. All writers reviewed and approved the manuscript. All authors contributed to the article and approved the submitted version.
